# Loss of biased signaling at a G protein-coupled receptor in overexpressed systems

**DOI:** 10.1371/journal.pone.0283477

**Published:** 2023-03-24

**Authors:** Angus Li, Samuel Liu, Rennica Huang, Seungkirl Ahn, Robert J. Lefkowitz

**Affiliations:** 1 Department of Medicine, Duke University Medical Center, Durham, North Carolina, United States of America; 2 Department of Biochemistry, Duke University Medical Center, Durham, North Carolina, United States of America; 3 Howard Hughes Medical Institute, Duke University Medical Center, Durham, North Carolina, United States of America; Indian Institute of Technology Kanpur, INDIA

## Abstract

G protein-coupled receptors (GPCRs) regulate cellular signaling pathways by coupling to two classes of transducers: heterotrimeric G proteins and β-arrestins. [Sarcosine^1^Ile^4^Ile^8^]-angiotensin II (SII), an analog of the endogenous ligand angiotensin II (AngII) for the angiotensin II type 1 receptor (AT_1_R), fails to activate G protein in physiologically relevant models. Despite this, SII and several derivatives induce cellular signaling outcomes through β-arrestin-2-dependent mechanisms. However, studies reliant on exogenous AT_1_R *overexpression* indicate that SII is a partial agonist for G protein signaling and lacks β-arrestin-exclusive functional specificity. We investigated this apparent discrepancy by profiling changes in functional specificity at increasing expression levels of AT_1_R using a stably integrated tetracycline-titratable expression system stimulated with AngII, SII, and four other AngII analogs displaying different signaling biases. Unbiased and G protein-biased ligands activated dose-dependent calcium responses at all tested receptor concentrations. In contrast, β-arrestin-biased ligands induced dose-dependent calcium signaling only at higher AT_1_R overexpression levels. Using inhibitors of G proteins, we demonstrated that both G_i_ and G_q/11_ mediated overexpression-dependent calcium signaling by β-arrestin-biased ligands. Regarding β-arrestin-mediated cellular events, the β-arrestin-biased ligand TRV026 induced receptor internalization at low physiological receptor levels insufficient for it to initiate calcium signaling. In contrast, unbiased AngII exhibited no relative preference between these outcomes under such low receptor conditions. However, with high receptor overexpression, TRV026 lost its functional selectivity. These results suggest receptor overexpression misleadingly distorts the bias of AT_1_R ligands and highlight the risks of using overexpressed systems to infer the signaling bias of GPCR ligands in physiologically relevant contexts.

## Introduction

GPCRs constitute the largest family of cell surface receptors in the mammalian genome and, owing to their regulation of a wide range of physiological processes, are the most common target of therapeutic drugs [1, 2]. Classically, GPCRs signal by activating heterotrimeric G proteins, which subsequently engage effector enzymes that modulate the production of second messengers [2]. In this paradigm, β-arrestins are recruited to activated and phosphorylated receptors and mediate desensitization by sterically hindering G protein coupling [3, 4] and serving as adaptors for receptor endocytosis [5]. However, β-arrestins can also interact with many signaling proteins, thereby acting as transducers and initiating β-arrestin-dependent signaling pathways [6].

Some GPCR ligands preferentially activate particular signaling pathways and as such are classified as “biased” agonists. Because different signaling pathways can trigger distinct physiological outcomes, biased agonists could serve as novel therapeutics with superior functional specificity [7, 8]. For example, β-arrestin-biased angiotensin II (AngII) receptor agonists promote cardioprotective signaling and contractility compared to conventional angiotensin receptor blockers but likewise antagonize deleterious G protein-mediated effects [9–11]. Consequently, they have been favorably evaluated for the treatment of long-term cardiomyopathy [12] in animal models and proposed as improved protectants against dysregulation of the renin-angiotensin-aldosterone system in severe COVID-19 disease [13].

[Sarcosine^1^Ile^4^Ile^8^]-angiotensin II (SII) is the first characterized biased AngII type 1 receptor (AT_1_R) agonist that specifically activates β-arrestin-mediated signaling pathways [14]. In multiple cellular models, including vascular smooth muscle cells and isolated cardiomyocytes, SII fails to induce G protein activation [11, 14–19]. Nevertheless, SII stimulation of the AT_1_R results in receptor internalization [14, 18], chemotaxis [16], positive inotropy [11], and activation of the extracellular signal-regulated kinases 1 and 2 (ERK1/2) and other cellular effectors through β-arrestin-2-dependent mechanisms [14, 17, 19–23]. Furthermore, the SII derivatives TRV023, TRV026, and TRV027 more potently trigger many of these responses while also not activating G protein and are considered to be β-arrestin-biased AT_1_R agonists of potential therapeutic interest [10].

Contrary to this extensive literature, several studies utilizing bioluminescence resonance energy transfer (BRET) and other experimental strategies that involve exogenous receptor overexpression indicate that SII and its derivatives lack specificity towards β-arrestin-mediated pathways and instead function as partial agonists for G protein signaling [24–26]. While BRET and similar approaches may facilitate direct and sensitive interrogation of the activation of different G protein isoforms [24, 26], alteration of receptor expression can also introduce system bias that perturbs functional selectivity independently of ligand bias [27]. Indeed, at increased receptor densities, numerous G_s_-coupled receptors additionally activate noncanonical phospholipase C pathways [28]. Similarly, the adenosine A_1_ receptor, which is classically G_i_-coupled, gains the ability to activate adenylyl cyclase through G_s_ [29].

Accordingly, we used here a stably integrated tetracycline-inducible titratable expression system to investigate the effects of AT_1_R expression on multiple signaling responses to various agonists in a single cell line. Through selective chemical inhibition, we also assessed the roles of different G protein subfamilies in mediating these activities. Our data illuminate how receptor expression acts orthogonally of ligand bias to generate distinct functional profiles at the AT_1_R and how overexpression of receptors at levels well above those normally found in tissues can lead to erroneous conclusions about bias in physiologic contexts.

## Results

### An inducible TetOn system allows robust and consistent modulation of AT_1_R expression

**Fig 1:**
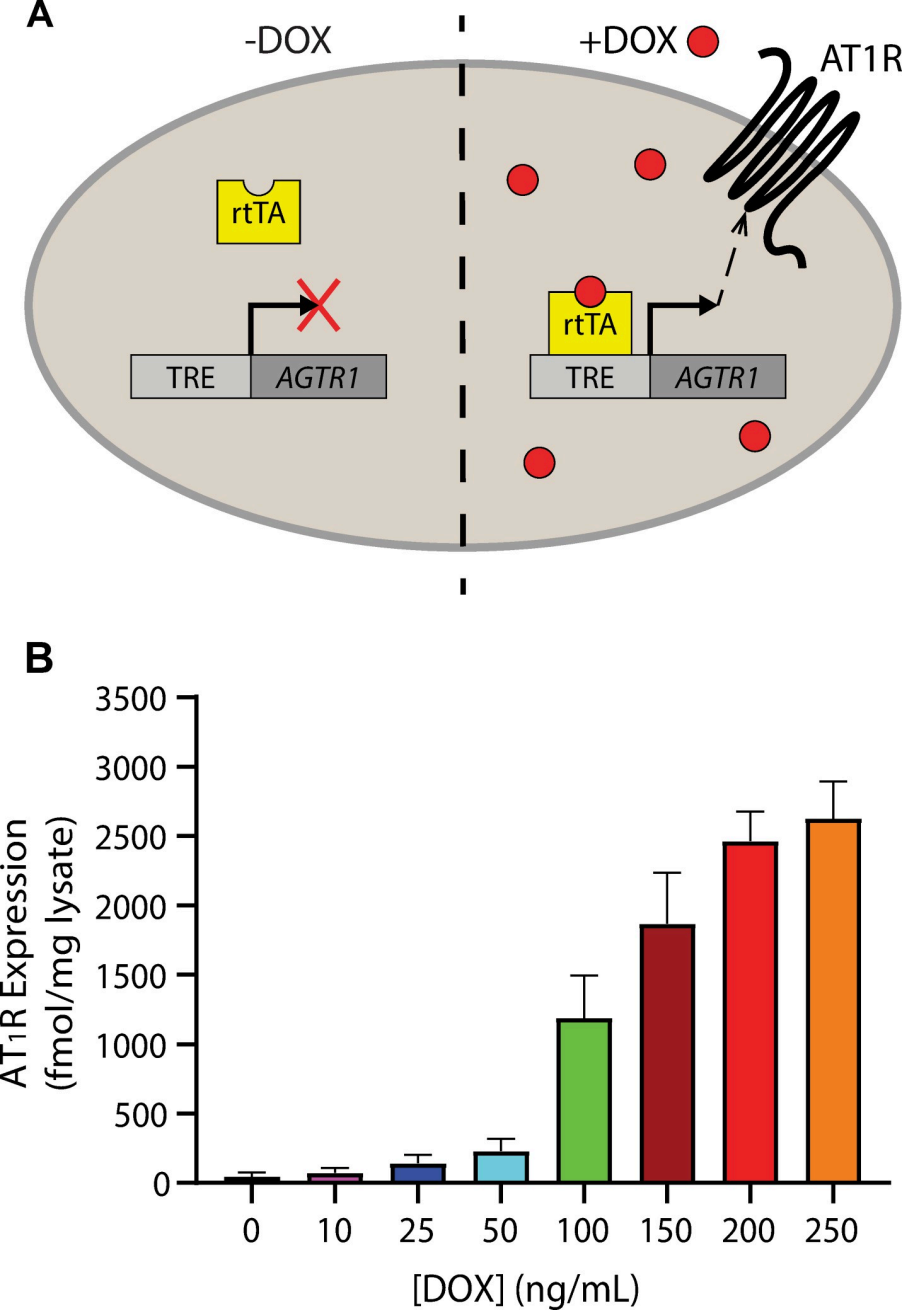
An inducible TetOn system facilitates consistent titration of AT_1_R expression in modified U2OS cells. (**A**) Schematic illustration of the U2OS-TetOn-AT_1_R system. Addition of doxycycline triggers binding of reverse tetracycline-controlled transactivator (rtTA) to tetracycline response element (TRE) in the promoter region of a sequence encoding the AT_1_R, thereby activating transcription and subsequent expression of the receptor in a titratable manner. (**B**) Doxycycline-induced receptor expression in U2OS-TetOn-AT_1_R cells. U2OS-TetOn-AT_1_R cells were incubated with indicated concentrations of doxycycline for 14 h. The level of receptor expression was determined by whole cell saturation binding of radiolabeled ligands and normalized to total protein content as determined by BCA assay. Values represent Mean ± SEM obtained from four independent experiments done in duplicate.

Biased agonism at the AT_1_R has been profiled in cellular systems with widely differing receptor levels, ranging from ~15 fmol/mg endogenous expression in vascular smooth muscle cells [19] to ~7.1 pmol/mg overexpression in stably transfected human embryonic kidney (HEK) 293 cells [25]. To investigate how such variations could affect signaling behaviors in the absence of confounding factors between cell lines, we sought to utilize a cellular system with consistently titratable AT_1_R expression. The creation of a stable tetracycline-inducible AT_1_R expression cell line using a TetOn system has been previously described [30]. Such systems consist of two elements–a constitutively expressed reverse tetracycline-controlled transactivator (rtTA) and a gene of interest controlled by an rtTA-dependent tetracycline response element (TRE)–and thereby enable titration of gene expression by treatment with variable amounts of doxycycline (Fig 1A) [31]. Using whole-cell saturation binding of the radiolabeled ligands [^3^H]-olmesartan and [^3^H]-AngII, we measured doxycycline-induced cell surface receptor expression in a U2OS-derived cell line stably expressing a TetOn system for the AT_1_R (hereafter referred to as U2OS-TetOn-AT_1_R). Cell surface receptor density was ~46 fmol/mg in the absence of doxycycline, likely due to constitutive leaky expression, but reached up to ~2.6 pmol/mg at a maximal doxycycline dosage of 250 ng/ml, thus confirming a suitably broad range of receptor levels ranging from near endogenous to more than 50-fold overexpression (Fig 1B).

### AT_1_R overexpression uniquely enables β-arrestin-biased agonists to initiate calcium signaling

**Fig 2:**
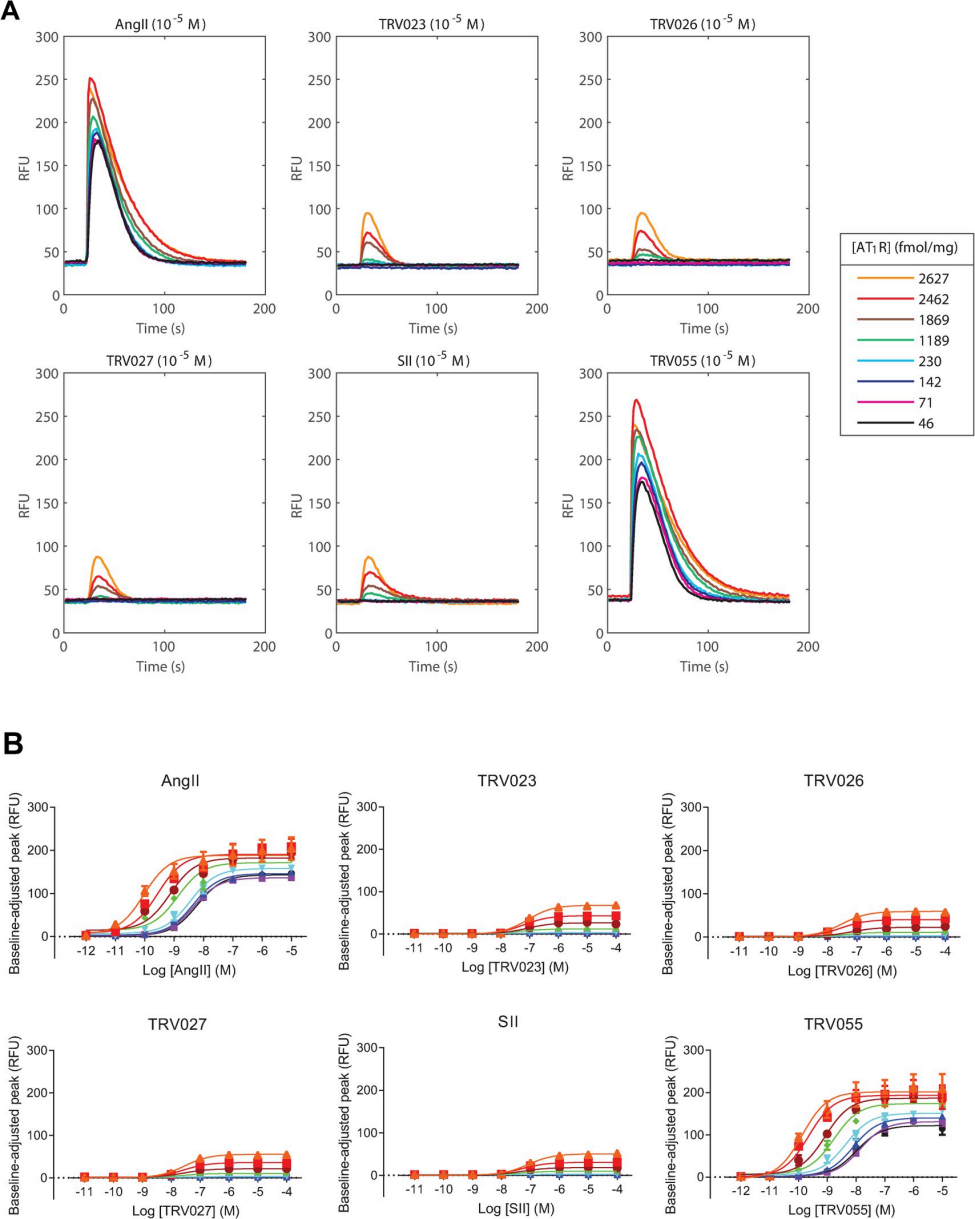
Calcium mobilization in response to unbiased and biased AT_1_R ligands at increasing levels of receptor expression. U2OS-TetOn-AT_1_R cells were incubated with appropriate concentrations of doxycycline for 14 h and stimulated with indicated ligands and concentrations. (**A**) Representative kinetic patterns for four independent experiments of calcium fluorimetric traces with 10 μM agonist stimulation at increasing levels of doxycycline-induced receptor expression. RFU, relative fluorescent unit. Different levels of surface AT_1_R expression are displayed at the right panel. (**B**) Dose-response of agonist-stimulated peak calcium fluorimetric signals at increasing levels of receptor expression. Each data point was generated by subtracting the average of the first 10 s as baseline signal and represents Mean ± SEM obtained from at least four independent experiments.

G protein activation through the AT_1_R induces phospholipase C (PLC) to produce inositol-1,4,5-triphosphate (IP_3_), rapidly triggering mobilization of calcium from intracellular stores [32]. Accordingly, a lack of these responses upon stimulation with SII or its derivatives is posed as evidence for these ligands’ inability to activate G proteins [10, 11, 14–16, 19]. Conversely, the presence of such responses in several overexpression-based studies has been presented as evidence that these ligands are partial agonists for G protein-mediated signaling [24, 25, 33]. We investigated this discrepancy by profiling cytosolic Ca^2+^ influx at different receptor expression levels following stimulation with each of the following ligands: unbiased AngII; the β-arrestin-biased agonists TRV023, TRV026, TRV027, and SII; or the G_q/11_-biased agonist TRV055 [25, 34, 35]. Cells treated with AngII or TRV055 exhibited dose-dependent Ca^2+^ responses at all assessed levels of receptor expression. Conversely, TRV023, TRV026, TRV027, or SII stimulation only triggered dose-dependent Ca^2+^ mobilization at doxycycline-induced receptor overexpression levels of at least ~1200 fmol/mg (Fig 2A and 2B). Together, these data indicate that in our inducible cell line, β-arrestin-biased AT_1_R agonists only gain the ability to initiate minimal Ca^2+^ mobilization at receptor concentrations at least 25-fold higher than basal constitutive levels of receptor expression. This low expression, by contrast, is sufficient to enable unbiased and G protein-biased agonists to activate such responses.

### Both G_i_ and G_q/11_ mediate overexpression-exclusive calcium signaling by β-arrestin-biased AT_1_R agonists

**Fig 3:**
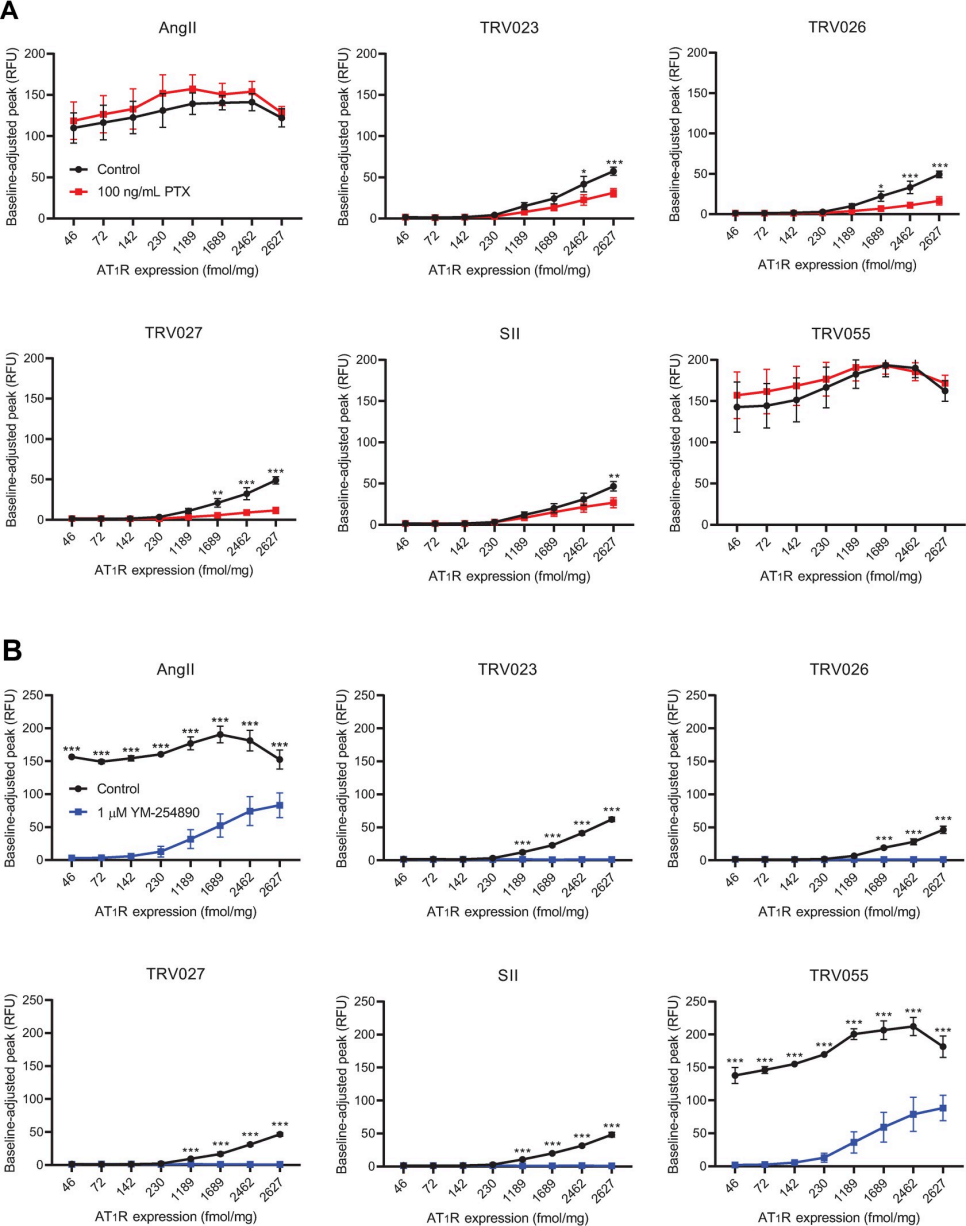
Calcium mobilization at increasing receptor levels upon chemical inhibition of G_i_ or G_q/11_ signaling. U2OS-TetOn-AT_1_R cells were incubated with appropriate concentrations of doxycycline for 14 h. Cells were pretreated with vehicle control or either (**A**) 100 ng/mL pertussis toxin (PTX) for 14 h for G_i_ inhibition or (**B**) 1 μM YM-254890 for 1 h for G_q/11_ inhibition prior to stimulation with 10 μM of indicated ligands. Each data point represents Mean ± SEM obtained from four independent experiments. Statistical significance was determined by ordinary two-way ANOVA and multiple comparisons testing between vehicle and inhibitor treatment groups at each level of AT_1_R expression, as described in Materials and Methods. *, p < 0.05; **, p < 0.01; ***, p < 0.001.

In physiological contexts, the AT_1_R primarily activates PLC and thus calcium signaling by coupling to heterotrimeric G proteins of the G_q/11_ family [36]. However, the AT_1_R additionally triggers some functional responses by activating G_i_ [16, 36], and Gβγ subunits released following dissociation of G_i_ family heterotrimers can also activate PLC [37]. In agreement with this general dual G protein signaling capability of the AT_1_R, some overexpression-dependent experimental approaches find SII to activate both G_q/11_ and G_i_ [24], while certain BRET biosensors even indicate a preference of SII and TRV027 to activate G_i_ more strongly than G_q/11_ [26]. We therefore sought to determine the contributions of the G_i_ and G_q/11_ families to *the overexpression-exclusive calcium signaling* induced by β-arrestin-biased AT_1_R agonists and to assess whether dependence on these G protein families differed for responses to unbiased and G protein-biased agonists. Pretreatment with the selective G_i_ family inhibitor pertussis toxin (PTX) [38] partially abolished calcium responses to all β-arrestin-biased agonists but had no effect on responses to AngII or TRV055 (Fig 3A). In contrast, the selective G_q/11_ family inhibitor YM-254890 [39, 40] fully eliminated all calcium signaling by β-arrestin-biased agonists and partially but robustly reduced responses elicited by AngII and TRV055 (Fig 3B). These data indicate that the overexpression-exclusive calcium signaling induced by β-arrestin-biased agonists is both partly mediated by G_i_ and entirely dependent on G_q/11_, implying that the two G protein families play non-additive roles in such responses and that some portion of calcium signaling depends on both G_i_ and G_q/11_. Indeed, this interpretation agrees with previously published findings that G_i_-mediated calcium signaling requires relief of PLC autoinhibition by active G_q/11_ [41] and that wholesale chemical inhibition of G_q/11_ activity abrogates such G_i_-dependent calcium responses [40]. Furthermore, the dependence of calcium responses to β-arrestin-biased agonists on both G_i_ and G_q/11_ differs sharply from the exclusive dependence of AngII and TRV055-induced calcium signaling on G_q/11_. Such findings corroborate published BRET assay data describing a relative preference for G_i_ activation over G_q/11_ by β-arrestin-biased agonists, albeit all at high levels of receptor expression [26].

### The β-arrestin-biased AT_1_R agonist TRV026 induces receptor internalization at low receptor densities while losing functional selectivity with high receptor overexpression

**Fig 4:**
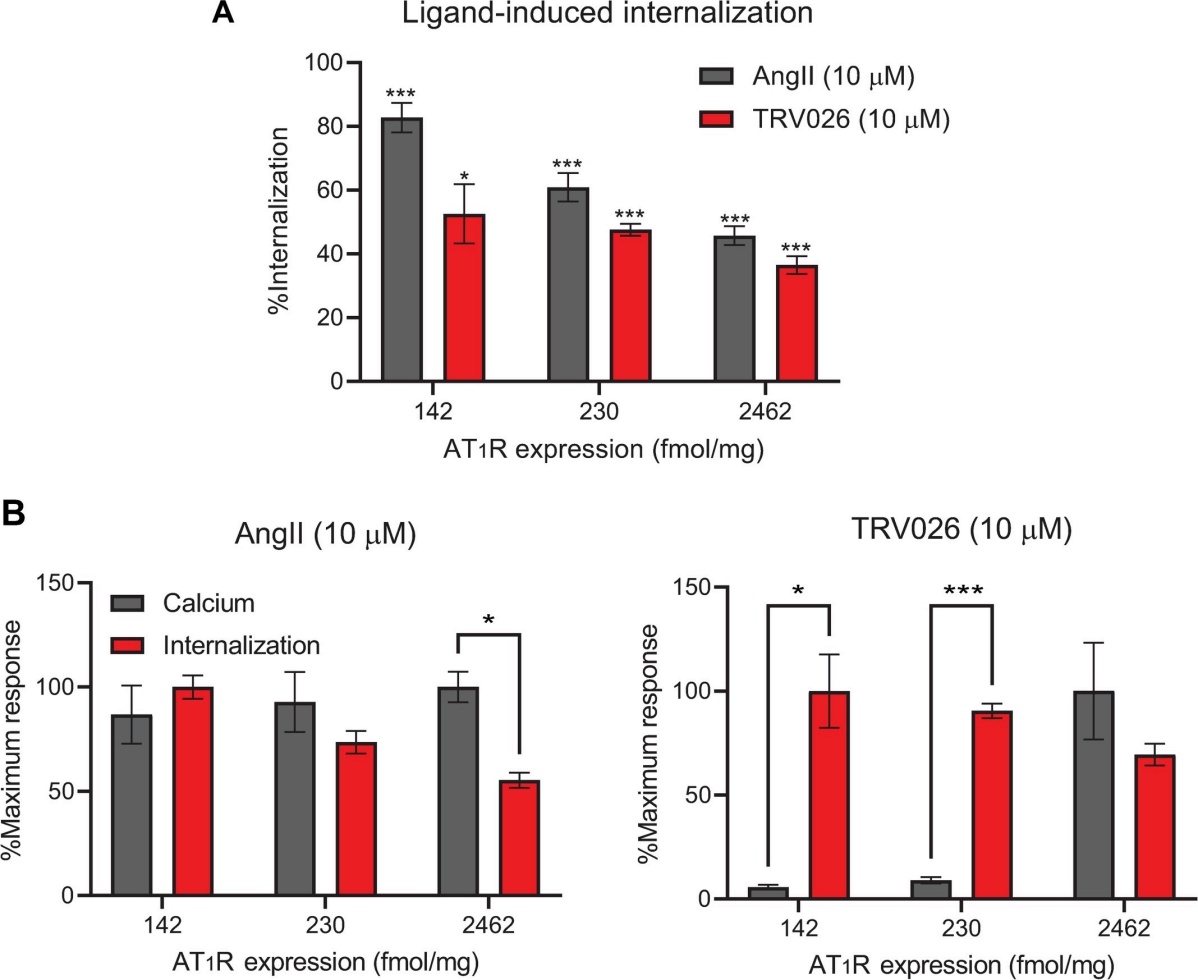
Agonist-induced receptor internalization at increasing levels of receptor expression. U2OS-TetOn-AT_1_R cells were incubated with appropriate levels of doxycycline for 14 h prior to stimulation with indicated ligands for 1 hr. The level of receptor was determined by radiolabeled ligand binding as described in Materials and Methods. (**A**) Ligand-induced percentage loss of cell surface receptor expression. Internalization was calculated as percent decreases in background-adjusted scintillation counts compared to the unstimulated, vehicle-treated condition for each individual experiment. Values represent Mean ± SEM obtained from four independent experiments. Statistical significance was determined by one-sample t-tests comparing each experimental group with a theoretical mean of zero percent internalization as described in Materials and Methods. *, p < 0.05; **, p < 0.01; ***, p < 0.001. (**B**) Comparison of relative calcium and receptor internalization responses. Calcium data were extracted from the dose-response experiments summarized in Fig 2A and 2B. All calcium and receptor internalization values were normalized per ligand as percentages of the maximal mean responses obtained in their respective assay formats, as described in Materials and Methods. Displayed values represent Mean ± SEM. Statistical significance was determined by two-way repeated measures ANOVA and multiple comparisons tests between normalized calcium and internalization responses at each tested receptor expression level as described in Materials and Methods. *, p < 0.05; **, p < 0.01; ***, p < 0.001.

In multiple cellular systems, AngII stimulation of the AT_1_R triggers functional outcomes through both G protein- and β-arrestin-2-dependent mechanisms. On the other hand, SII-induced responses appear to be independent of G protein activation [14, 17–19, 23], although SII and its β-arrestin-biased derivatives are less efficacious than AngII, even for β-arrestin-dependent responses [25, 35]. Therefore, we selected a β-arrestin-biased ligand, TRV026, and examined its competency to induce receptor internalization, a process mediated by β-arrestins [5, 42, 43], without activating G protein in our U2OS-TetOn-AT_1_R system. We titrated AT_1_R expression to 142 fmol/mg and 230 fmol/mg, concentrations at which β-arrestin-biased agonists did not activate G protein-mediated calcium signaling (Figs 2A, 2B, 3A and 3B). Under these conditions, stimulation with both AngII and TRV026 triggered substantial receptor internalization, measured as percentage loss of cell surface receptor expression (Fig 4A). Combined with the calcium response data, these results suggest that at low receptor densities, β-arrestin-biased AT_1_R agonists initiate functional responses independent of G protein activation. Such evidence recapitulates findings in vascular smooth muscle cells [19], left ventricular tissue samples [9], isolated cardiac myocytes, and Langendorff-perfused hearts [17] while contrasting with data obtained from some overexpressed receptor systems [24, 26]. Additionally, agonist-induced receptor internalization *percentage* decreased with increasing receptor expression (Fig 4A), as would be expected [35].

At high expression levels, numerous GPCRs gain the ability to activate noncanonical effectors while also maintaining canonical pathways [28, 29]. Such behavior contradicts conventional receptor theory, which postulates that all responses should increase directly and proportionally with receptor expression [44]. To examine whether a similar pattern might describe the activity of β-arrestin-biased agonists at the AT_1_R, we normalized the extent of calcium influx and receptor internalization to their respective maximum responses to enable direct comparison between these two functional outcomes at different receptor concentrations. TRV026 was effectively completely selective for the receptor internalization process over calcium signaling at low levels of receptor expression, but this preference was lost at higher receptor densities. Behaviors induced by AngII did not follow this trend; normalized calcium and internalization responses did not differ from each other at low levels of receptor expression, while calcium signaling was slightly favored over induction of receptor internalization with high receptor overexpression (Fig 4B). This apparent bias, however, is likely reflective of the differing natures of second messenger and receptor internalization assays. According to classical models of GPCR function, receptor overexpression should drive higher overall levels of G protein activation, leading to elevated or saturated readouts of second messengers. Conversely, a relative paucity of β-arrestin relative to receptor should decrease the proportion of receptor that is internalized, even though the total number of internalized receptor molecules is increased [35, 45]. Together, these results indicate that at low receptor levels approaching those that are physiologically relevant, TRV026 exhibits a unique selectivity for β-arrestin-mediated functional outcomes. Such a selectivity is not shared by AngII and is lost at high receptor overexpression, which drives a non-physiologic gain of function for TRV026 to activate G protein-mediated signaling.

## Discussion

Biased agonism for GPCR signaling is an attractive goal for drug discovery efforts, because effecting functional outcomes with finer specificity could improve and expand therapeutic options in many disease systems [7, 8, 27]. β-Arrestin-biased agonism at the AT_1_R holds therapeutic potential, owing to the deleterious consequences of excessive G protein-mediated signaling and, conversely, the positive physiological effects of β-arrestin-dependent pathways through the AT_1_R [9, 12, 13, 46, 47]. Accordingly, establishing appropriate systems to test the signaling behavior of different AT_1_R ligands is important for both basic investigation of biased agonism and evaluation of potential drug candidates. Findings in overexpressed systems of G protein activation by putatively β-arrestin-biased AT_1_R agonists [24–26, 33] raise important questions about the relevance and technical merits of different experimental strategies to profile GPCR signaling. Now, our study demonstrates that receptor overexpression markedly alters the specificity of β-arrestin-biased AT_1_R agonists, leading to gain of functions (Figs 2A, 2B, 3A, 3B and 4B) not observed in physiologically relevant systems [9, 11, 17, 19]. These data mirror previous findings with other GPCRs and their downstream effectors [28, 29], and they caution against the uncritical use of overexpression systems to characterize GPCR activity. Such systems are powerful tools for exploration and hypothesis generation, but the data they produce must be interpreted carefully and validated in more physiologically representative models.

Investigations using cell lines modified by CRISPR/Cas9-mediated genetic knockout together with application of chemical inhibition have reported that the AT_1_R and several other GPCRs fail to activate cellular signaling responses in the absence of active G proteins. Based on such results, the authors of these studies argue that GPCRs mediate downstream processes exclusively through G protein activation [48]. However, genetic deletion of important regulatory proteins is also likely to cause compensatory and inconsistent signal rewiring and is therefore an imperfect strategy to ascertain the roles of such proteins in less heavily engineered systems. Indeed, other studies have shown that the AT_1_R regulates functional responses through both G protein-dependent and independent mechanisms [14, 19, 23, 49], the relative predominances of which may shift with changes in receptor expression. Furthermore, the system bias induced by differential expression of effectors is likely to be strongest with the high degrees of overexpression or knockdown found in engineered experimental systems, though it is even recognized that in physiological contexts, a GPCR ligand can exhibit varying functional selectivity across tissues, owing to differing expression levels of signal transducers [27]. Our own data (Fig 4A and 4B) corroborate that β-arrestin-mediated receptor internalization persists in the absence of G protein activation [48], while also further demonstrating that perturbed expression of signaling components greatly alters signaling specificity, even in the same cellular system.

What molecular mechanisms might drive perturbations in functional specificity upon receptor overexpression? Recent population-based methods such as double electron-electron resonance (DEER) spectroscopy show that receptors under various conditions are not adequately represented by singular static structures but rather constitute heterogeneous ensembles of conformational states [34]. Such conformational heterogeneity provides a potential mechanistic framework to explain changes in observable functional selectivity with receptor expression. Conformational states with a propensity to activate G protein-mediated signaling could represent a small but nonzero proportion of β-arrestin-biased agonist-bound AT_1_R ensembles. At near-physiologic levels of receptor expression, this population would contribute negligibly to the overall cellular signaling response, leading to a lack of G protein activation. By contrast, overexpression would increase the total number of β-arrestin-biased agonist-bound receptor molecules that adopt these G protein-favoring conformational states, thereby triggering measurable amounts of G protein activation. These “numbers game” mechanisms are also compatible with the ability of β-arrestins to uncouple or desensitize G protein-mediated signaling [3, 4]; overexpression of the receptor combined with saturating dosing could allow the number of β-arrestin-biased agonist-bound AT_1_R complexes to exceed the endogenous population of β-arrestins, leading to appreciable G protein activation that would be fully suppressed under physiological conditions. Indeed, in similar inducible expression systems, β-arrestin overexpression counteracts increases in G protein-mediated responses following receptor overexpression [30].

Indeed, in our study, a concentration of the G_q/11_ family inhibitor YM-254890 that fully abolishes calcium responses to β-arrestin-biased agonists at all AT_1_R expression levels and to AngII and TRV055 at low receptor levels only partially eliminates calcium signals induced by the latter group of agonists with high receptor overexpression (Fig 3B). These results imply that for a given receptor concentration, the number of total AT_1_R molecules capable to activate G protein is much higher under stimulation with unbiased or G protein-biased agonists than with β-arrestin-biased agonists. Thus, at high receptor levels, unbiased and G protein-biased agonists at their saturating concentrations activate G protein too robustly to be completely abolished with YM-254890 at the concentration utilized in our study. As a further possibility for the partial inhibitory effect of YM-254890 at the saturating concentrations of unbiased or G protein-biased agonists, the AT_1_R has been reported to signal additionally through G_12/13_ [26] and possibly G_s_. The activation of these noncanonical G proteins by the AT_1_R could theoretically initiate calcium signaling either through free G protein subunit activity or production of second messengers. Such activities remain uncharacterized.

We conclude that receptor overexpression can cause signaling outcomes to diverge from those observed in physiologically relevant systems. This results in loss of ligand bias observed at more physiological levels of receptor expression and presents an important obstacle to drug development unless more widely appreciated.

## Materials and methods

### Antibodies and reagents

AngII, TRV023, TRV026, TRV027, SII, and TRV055 were synthesized by GenScript. [^3^H]-olmesartan and [^3^H]-AngII were from American Radiolabeled Chemicals. Candesartan was from Alfa Aesar. PTX was from List Biological Laboratories. YM-254890 was from Cayman Chemical Company. FLIPR Calcium 6 Assay dye was from Molecular Devices.

### Cell culture

U2OS-TetOn-AT_1_R cells were generated as previously described [30]. Briefly, U2OS cells were transfected with pTet-On and subjected to selection with 500 μg/ml G418 for 3 weeks. Stable clones were then transfected with pTRE2-hyg-HA-AT_1_R and subjected to selection with 200 μg/ml hygromycin for 3 weeks. Positive clones with low background and ability to express AT_1_R upon doxycycline treatment were maintained in minimal essential medium with 100 μg/μl G418 and 100 μg/μl hygromycin supplemented with 10% fetal bovine serum, 1% penicillin-streptomycin, and 2 mM GlutaMAX (Gibco).

### Radioligand binding and receptor internalization measurement

U2OS-TetOn-AT_1_R cells grown at ~165,000 cells/well in poly-D-lysine-coated 12-well plates were treated with doxycycline for 14 h. For receptor expression measurements, media was removed from plates and the cells were incubated in minimal essential medium containing 20 nM [^3^H]-AngII or 50 nM [^3^H]-olmesartan for 1.5 h. Nonspecific binding was measured by simultaneous addition of 200 μM candesartan to radioligand-containing media. Cells were washed with ice-cold PBS containing MgCl_2_ and CaCl_2_ and lysed with a solution of 0.1% SDS and 0.5 N NaOH. Radioactivity of cell lysates was measured by scintillation counting. Total protein was determined using the Pierce BCA protein assay (Thermo Scientific) and used to convert scintillation counts to cell surface AT_1_R concentration in Radioactivity Calculator (GraphPad Software). The full protocol for these experiments is publicly archived at dx.doi.org/10.17504/protocols.io.ewov1oxe2lr2/v1. For receptor internalization measurements, experiments were performed as above, except that cells were stimulated with unlabeled ligand for 1 hr and washed with an ice-cold acid stripping solution consisting of 0.2 M acetic acid and 0.5 M NaCl adjusted to pH 2.5 for 5 min prior to addition of radiolabeled ligand, and [^3^H]-AngII was used for all measurements. The extent of ligand-induced receptor internalization was determined as loss of cell surface receptor expression upon stimulation with ligands. The full protocol for these experiments is publicly archived at dx.doi.org/10.17504/protocols.io.x54v9dz91g3e/v1.

### Calcium fluorimetry

U2OS-TetOn-AT_1_R cells grown at 15,000 cells/well in poly-D-lysine-coated 96-well black well plates were treated with doxycycline for 14 h. For studies of PTX inhibition of calcium signaling, cells were treated with 100 ng/ml PTX or vehicle concurrent with addition of doxycycline. Media was removed from plates and the cells were incubated with calcium-sensitive fluorescent dye from the FLIPR Calcium 6 assay kit for 2 h according to the manufacturer’s instructions. For studies of YM-254890 inhibition of calcium signaling, cells were treated with 1 μM YM-254890 or vehicle for 1 h. Fluorescence was measured in a FlexStation 3 microplate reader (Molecular Devices) for 3 min according to the manufacturer’s instructions. Cells were stimulated with ligand 20 s after starting fluorescence measurements. Fluorescence measurements for each well were adjusted to baseline by subtracting the average signal from the first 10 s of measurement. All calcium responses were quantified as baseline-adjusted peak fluorescent signal. The full protocol for these experiments is publicly archived at dx.doi.org/10.17504/protocols.io.3byl4jdmjlo5/v1.

### Graphing and statistical analysis

Raw calcium assay data were analyzed and graphed using MATLAB R2020b (Mathworks). All other data were graphed using Prism 9.4 (GraphPad Software). Calcium data were fitted to a log(agonist) versus response model with three parameters (baseline, span, and EC_50_) in Prism 9.4. Statistical analyses were also performed in Prism 9.4. Calcium dose response data were subjected to ordinary two-way analysis of variance (ANOVA) and post-hoc multiple comparisons tests of peptide agonist dose effects within groups of different receptor expression levels. A statistically significant difference of calcium response values between at least one agonist dosage and the minimum tested dose, as assessed by Dunnett’s multiple comparisons tests, was interpreted to indicate a dose-dependent response. The effects of PTX and YM-254890 treatment were analyzed using ordinary two-way analysis of variance (ANOVA) and post-hoc Šidák’s multiple comparisons tests of inhibitor effects within groups of different receptor concentrations. Receptor internalization responses were subjected to one-sample two-tailed t-tests comparing each experimental group to a null hypothesis mean of zero percent internalization, with significance interpreted to indicate ligand-induced internalization. To enable statistical testing between different functional readouts, calcium and internalization responses were normalized, per ligand, to percent of the maximal mean response in their respective assay format across all receptor levels. They were then compared at different receptor concentrations using two-way repeated measures ANOVA and post-hoc Šidák’s multiple comparisons tests at each tested receptor expression level.
